# Outcome of bimodality definitive chemoradiation does not differ from that of trimodality upfront neck dissection followed by adjuvant treatment for >6 cm lymph node (N3) head and neck cancer

**DOI:** 10.1371/journal.pone.0225962

**Published:** 2019-12-03

**Authors:** Wan-Yu Chen, Tseng-Cheng Chen, Shih-Fan Lai, Tony Hsiang-Kuang Liang, Bing-Shen Huang, Chun-Wei Wang

**Affiliations:** 1 Division of Radiation Oncology, Department of Oncology, National Taiwan University Hospital, Taipei, Taiwan; 2 Graduate Institute of Clinical Medicine, National Taiwan University College of Medicine, Taipei, Taiwan; 3 Cancer Research Center, National Taiwan University College of Medicine, Taipei, Taiwan; 4 Department of Otolaryngology, National Taiwan University Hospital and National Taiwan University, College of Medicine, Taipei, Taiwan; 5 Department of Radiation Oncology, Chang Gung Memorial Hospital and Chang Gung University, Taoyuan, Taiwan; 6 Department of Radiology, College of Medicine, National Taiwan University, Taipei, Taiwan; University of Wisconsin, UNITED STATES

## Abstract

Currently, data regarding optimal treatment modality, response, and outcome specifically for N3 head and neck cancer are limited. This study aimed to compare the treatment outcomes between definitive chemoradiotherapy (CCRT) to the neck and upfront neck dissection followed by adjuvant CCRT. Ninety-three N3 squamous cell carcinoma head and neck cancer patients were included. Primary tumor treatment was divided to definitive CCRT (CCRT group) or curative surgery followed by adjuvant CCRT (surgery group). Neck treatment was also classified into two treatment modalities: definitive CCRT to the neck (CCRT group) or curative neck dissection followed by adjuvant CCRT (neck dissection group). Overall, the 2-year overall survival (OS), local recurrence-free survival (LRFS), regional recurrence-free survival (RRFS), and distant metastasis-free survival (DMFS) were 51.8%, 47.3%, 45.6%, and 43.6%, respectively. In both oropharyngeal cancer and nonoropharyngeal cancer patients, in terms of OS, LRFS, RRFS or DMFS no difference was noted regarding primary tumor treatment (CCRT vs. surgery) or neck treatment (CCRT vs. neck dissection). In summary, N3 neck patients treated with definitive CCRT may achieve similar outcomes to those treated with upfront neck dissection followed by adjuvant CCRT. Caution should be made to avoid overtreatment for this group of patients.

## Introduction

Currently, data regarding optimal treatment modality, response, and outcome specifically for N3 head and neck cancer are limited. Most studies included a combination of N2 and N3 head and neck cancers, with only approximately 10–15% of N3 patients in prospective clinical trials[1–4] or retrospective studies[4, 5]. Planned neck dissection after definitive chemoradiotherapy (CCRT) can be omitted, and salvage post-RT neck dissection can be performed only in incomplete response to CCRT[3, 6]. However, some physicians choose neck dissection as primary treatment because of concerns for poor radiation response of bulky necrotic lymph nodes, anatomical change of bulky lymph nodes during radiation, and avoidance of post radiation neck dissection. For N3 head and neck cancer, there is limited data regarding whether direct neck dissection or definitive CCRT to the neck should be performed. This study aimed to compare the treatment outcomes between definitive CCRT to the neck and upfront neck dissection followed by adjuvant CCRT for N3 head and neck cancer patients.

## Materials and methods

### Patients and treatments

The retrospective study protocol was approved by the Research Ethics Committee of National Taiwan University Hospital (NTUH: 201707061RINB) and IRB approved that patient consent was waived. All patient data were anonymized before researchers gained access. Between 2002 and 2015, 93 N3 (>6 cm, American Joint Committee on Cancer 7th edition) squamous cell carcinoma head and neck cancer patients with no distant metastasis who received curative treatment at National Taiwan University Hospital were included in this study. Nodal dimensions were defined by magnetic resonance imaging (MRI). The median diameter of confluent neck LNs was 7.5 cm (range 6–10). Among the 93 patients, 76 (81.7%) received induction chemotherapy, which included the following regimens: PF (cisplatin + 5-FU), EPF (Erbitux + PF), APF (Avastin + PF), TPF (Taxotere + PF), ATPF (Avastin + TPF), MEPFL (mitomycin, epirubicin, cisplatin, fluorouracil, and leucovorin), intra-arterial (IA) MPA (mitomycin, cisplatin, Avastin), IA-MTPF (mitomycin, Taxotere, cisplatin, 5FU), IA-MATPF (MTPF + Avastin), or their combinations. For patients receiving induction chemotherapy, the median cycles received were 2 (range, 1–8). The overall response rate to induction chemotherapy was 68%. Curative treatments were categorized into options 1–3 as follows: 1) definitive CCRT to primary tumor and neck; 2) curative surgery for primary tumor and the neck followed by adjuvant CCRT; and 3) curative neck dissection followed by definitive CCRT for primary tumor and adjuvant CCRT for the neck. The treatments were summarized in the S1 Fig. Curative surgery for primary tumor comprised of wide tumor excision with flap reconstruction if necessary. Curative neck dissection includes modified radical neck dissection for bulky neck nodes with or without contralateral neck dissection at the discretion of the treating physician. Definitive CCRT irradiation dose was 70 Gy in 33–35 fractions, which was delivered concurrently with weekly 40 mg/m^2^ cisplatin. Sixty-seven (72%) patients completed all therapy. The median cycle of weekly cisplatin was 6 (range, 3–7) and 70 patients (75%) received cumulative dose of concurrent weekly cisplatin greater or equal to 200 mg/m^2^. Adjuvant RT dose was set to 60–66 Gy in 30–33 fractions.

Patients were routinely assessed 3–4 months after the completion of the treatment through clinical examination, chest X-ray, and head and neck MRI. For patients who received definitive CCRT, neck dissection was not routinely performed. Response evaluation in this study was done by both clinically local examination and MRI. Complete response was defined by undetectable primary tumor or shrinkage of neck lymph nodes to less than 1cm in short axis on T2 weighted and T1 weighted with contrast medium MRI. Salvage neck dissection or primary tumor excision was considered only if an incomplete response occurred.

### Immunohistochemical analysis of p16

Primary tumor sections of 4 μm thickness were deparaffinized and pretreated for antigen retrieval through autoclave heating (121°C) in 10 mM sodium citrate buffer (pH 6.0) for 10 min. These sections were blocked for endogenous peroxidase activity with 3% H2O2 in methanol for 10 min and then washed in phosphate-buffered saline. Thereafter, the sections were immersed in UltraVision Protein Block (Thermo Fisher Scientific, Fremont, LA, USA) for 10 min, covered with a primary rabbit monoclonal antibody specific for p16 (clone: EP1215Y, Epitomics, Abcam Company, Burlingame, CA, USA), and incubated for 1 h at room temperature. Immunoreactions were performed using the UltraVision Quanto Detection System HRP DAB (Thermo Fisher Scientific, Fremont, LA, USA). Immunohistochemical evaluation of p16 in oropharyngeal cancer specimens was based on the intensity and extent of nuclear and cytoplasmic reactivity. Positive p16 expression was defined as strong and diffuse nuclear and cytoplasmic staining in 70% or more of the tumor cells.

### Statistical analysis

Comparison of proportions across groups was performed using Chi-squared test or Fisher’s exact test when number <5. Unpaired Student's t test was used to compare parametrically distributed continuous data. The following endpoints were used for assessment: overall survival (OS), local recurrence-free survival (LRFS), regional recurrence-free survival (RRFS), and distant metastasis-free survival (DMFS). These endpoints were measured from the day of diagnosis. Survival curves were estimated via the Kaplan-Meier method. Univariate and multivariate analyses were performed with log-rank test and Cox regression, respectively. A two-sided p value <0.05 was considered statistically significant. Statistical analysis was performed with SPSS 19.0.

## Results

Table 1 shows the patients characteristics. The primary tumor sites included the oropharynx (n = 49) and nonoropharynx (n = 44; 26 hypopharynx, 14 oral cavity, and 4 larynx). The median smoking pack-year is 30 (range, 0–80). Patients with oropharyngeal malignancy were associated with more T1/T2 tumors (p = 0.030). Primary tumor treatment was divided to definitive CCRT (CCRT group; treatment options 1+3) or curative surgery followed by adjuvant CCRT (surgery group; treatment option 2). The oropharyngeal group had more patients receiving definitive CCRT to primary tumor sites (p = 0.030). Neck treatment was also classified into two treatment modalities: definitive CCRT to the neck (CCRT group; treatment option 1) or curative neck dissection followed by adjuvant CCRT (neck dissection group; treatment option 2+3). The oropharyngeal group had more patients receiving definitive CCRT to the neck (p = 0.000). In addition, patients who received curative operation to primary tumors, compared to definitive CCRT to primary tumors, were associated with more advanced T3/T4 tumors (p = 0.019), better performance status ECOG 0 (p = 0.023), and more ono-oropharyngeal cancer (p = 0.000). At presentation, 45% of nodes were considered unresectable. Patients who received curative neck dissection, compared to definitive CCRT to neck, were associated with better performance status ECOG 0 (p = 0.015) and ono-oropharyngeal cancer (p = 0.000). In our study, neck dissection was performed in 34 patients (36.3%). Among patients who received neck dissection, 30 out of 34 patients (88%) had pathological positive ECE. Clinical ECE was observed in 80 out of 93 patients (86%) according neck MRI. In addition, matted nodes (defined as three nodes abutting one another with loss of intervening fat plane) [7] prevalence rate was 62%. Patients with matted nodes had inferior DMFS (p = 0.015).

**Table 1:** Patient characteristics.

Characteristics	All patients No. (%) (N = 93)	Oropharynx No. (%) (N = 49)	Non- Oropharynx No. (%) (N = 44)	P value
Gender				
Male	89 (95.7)	48 (98.0)	41 (93.2)	
Female	4 (4.3)	1 (2.0)	3 (6.8)	0.341
Age (years old) (median, range)	52 (34–78)	53 (34–78)	51.5 (35–78)	0.870
T classification				
T1/T2	32 (34.4)	22 (44.9)	10 (22.7)	
T3/T4	61 (65.6)	27 (55.1)	34 (77.3)	0.030
Primary tumor treatment				
CCRT	71 (76.3)	45 (91.8)	26 (59.1)	
Surgery	22 (23.7)	4 (8.2)	18 (40.9)	0.000
Neck treatment				
CCRT	59 (63.4)	41 (83.7)	18 (40.9)	
Neck dissection	34 (36.6)	8 (16.3)	26 (59.1)	0.000
Radiotherapy				
Definitive to both primary and neck (option 1)	59 (63.4)	41 (83.7)	18 (40.9)	
Adjuvant (option 2)	22 (23.7)	4 (8.2)	18 (40.9)	
Definitive to primary and adjuvant to neck (option 3)	12 (12.9)	4 (8.2)	8 (18.2)	0.000
Induction chemotherapy				
No	17 (18.3)	5 (10.2)	12 (27.3)	
Yes	76 (81.7)	44 (89.8)	32 (72.7)	0.058
P16 positive rates		55%	14%	0.005

Abbreviation: CCRT = concurrent chemoradiation

Among patients who received definitive CCRT to primary tumor sites, oropharyngeal cancer patients had higher complete response (CR) rate than nonoropharyngeal cancer patients. A total of 37 (82.2%) and 19 (73.1%) patients had oropharyngeal and nonoropharyngeal cancer, respectively. The number (rate) of patients who achieved partial response (PR) was 8 (17.8%) and 7 (26.9%) in those with oropharyngeal and nonoropharyngeal cancer, respectively (p = 0.000). For patients who received definitive CCRT to the neck, the number of patients with oropharyngeal and nonoropharyngeal cancer who achieved CR were 31 (75.6%) and 12 (66.7%), respectively, and those who achieved PR were 10 (24.4%) and 6 (33.3%), respectively (p = 0.000). A total of 7 (22.6%) and 3 (25%) patients with oropharyngeal cancer and nonoropharyngeal cancer developed regional recurrence after CR was achieved post definitive neck CCRT, respectively.

The median follow-up time for all patients was 21.1 months (range, 6.9–105.4 months). The median follow-up time for censored patients or survivors was 41.8 months (range, 10.6–105.4 months; IQR: 23.4–73.2 months). Overall, the 2-year OS, LRFS, RRFS, and DMFS were 51.8%, 47.3%, 45.6%, and 43.6%, respectively. For all patients combined, neck treatment (CCRT vs. neck dissection) did not affect 2-yr OS (55.5% vs. 46.4%; p = 0.236, S2 Fig), LRFS (47.9% vs. 46.5%; p = 0.419, S2 Fig), RRFS (45.2% vs. 46.7%; p = 0.854, S2 Fig) or DMFS (49.2% vs. 34.2%; p = 0.172, S2 Fig).

**Fig 1:**
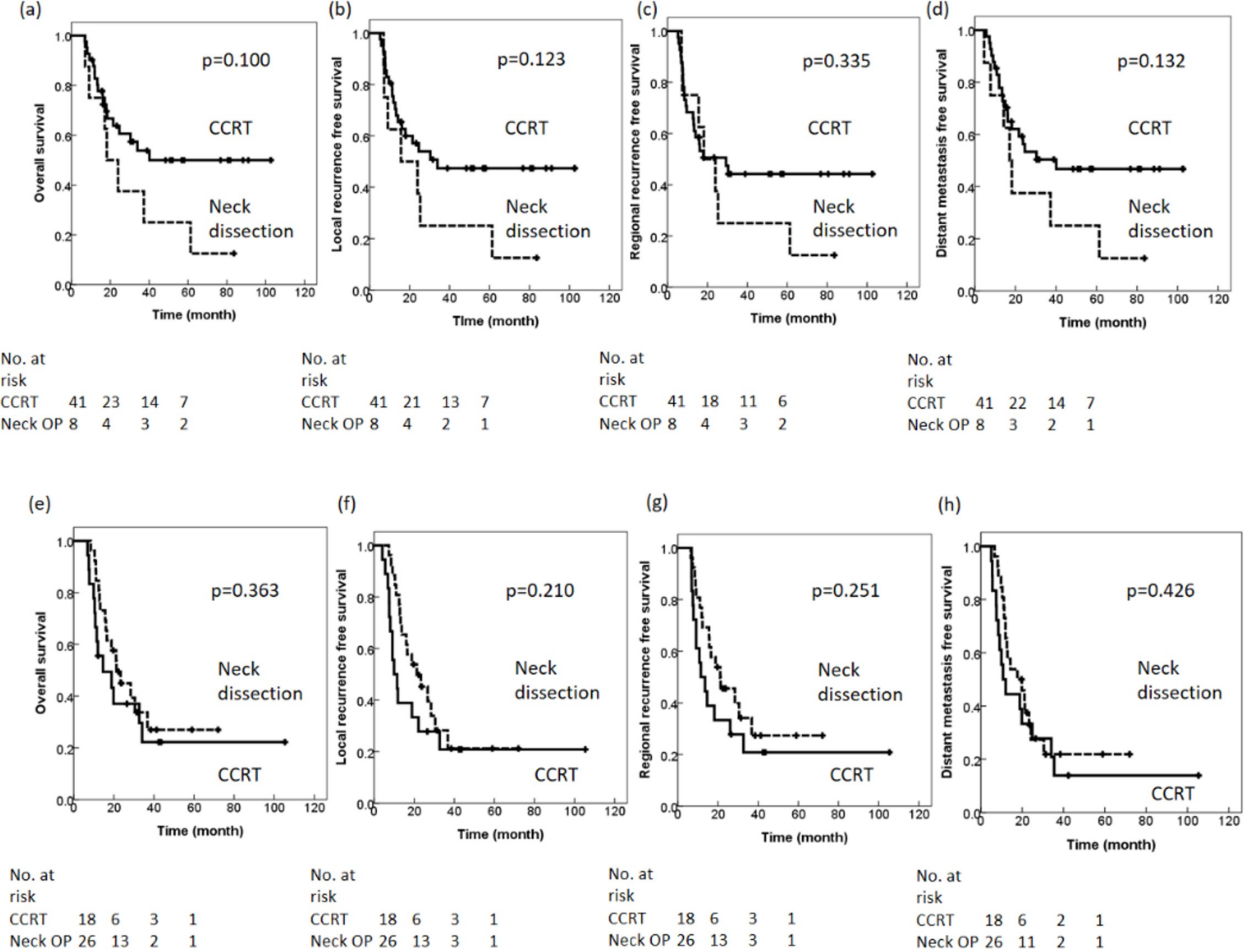
Survival curve. (a) OS, (b) LRFS, (c) RRFS, and (d) DMFS for oropharyngeal cancer patients. (e) OS, (f) LRFS, (g) RRFS, (h) and DMFS for nonoropharyngeal patients.

Univariate and multivariate analyses for survival rate in oropharyngeal cancer patients are summarized in Table 2. In oropharyngeal cancer patients, in terms of OS, no difference was noted regarding primary tumor treatment (Surgery vs. CCRT) (HR: 0.607; 95% CI: 0.123–3.000; p = 0.540) or neck treatment (neck dissection vs. CCRT) (HR: 2.199; 95% CI: 0.522–9.256; p = 0.283). Advanced T3/T4 stage was associated with worse OS (HR: 3.337; 95% CI: 1.312–8.488; p = 0.011). The 2-year OS rate for definitive CCRT to the neck (CCRT group) or curative neck dissection followed by adjuvant CCRT (neck dissection group) was 57.4% and 37.5%, respectively (Fig 1A). For LRFS, no difference was noted in terms of primary tumor treatment (surgery vs. CCRT) (HR: 0.446; 95% CI: 0.079–2.536; p = 0.363) or neck treatment (neck dissection vs. CCRT) (HR: 2.689; 95% CI: 0.448–16.145; p = 0.280). The 2-year LRFS rate for definitive CCRT to the neck (CCRT group) or curative neck dissection followed by adjuvant CCRT (neck dissection group) was 53.9% and 37.5%, respectively (Fig 1B). For RRFS, no difference was noted in terms of neck treatment (neck dissection vs. CCRT) (HR: 1.284; 95% CI: 0.270–6.115; p = 0.754). The 2-year RRFS for definitive CCRT to the neck (CCRT group) or curative neck dissection followed by adjuvant CCRT (neck dissection group) were 50.6% and 37.5%, respectively (Fig 1C). For DMFS, no difference was noted in terms of primary tumor treatment (surgery vs. CCRT) (HR: 0.706; 95% CI: 0.150–3.322; p = 0.660) or neck treatment (neck dissection vs. CCRT) (HR: 1.962, 95% CI: 0.503–7.660; p = 0.322). Advanced T3/T4 stage was associated with worse DMFS (HR: 3.307; 95% CI: 1.289–7.157; p = 0.011). The 2-year DMFS rate for definitive CCRT to the neck (CCRT group) and curative neck dissection followed by adjuvant CCRT (neck dissection group) was 56.3% and 37.5%, respectively (Fig 1D). Among 49 oropharyngeal cancer patients, 20 patients had adequate remaining pathology samples for IHC stain. Nine patients (45%) were p16+ and 11 patients (55%) were p16-. For 44 non-oropharyngeal cancers, 35 patients were tested for p16. However, only 5 patients (14%) were p16+. Significant differences in the 2-year OS (77.8% vs 45.5%, p = 0.009, respectively), 2-year LRFS (77.8% vs 45.5%, p = 0.009, respectively), 2-year RRFS (77.8% vs 27.3%, p = 0.002, respectively), and 2-year DMFS (77.8% vs 36.4%, p = 0.007, respectively) were observed between patients with HPV+ and HPV− oropharyngeal cancer.

**Table 2:** Univariate and multivariate analysis for survival in oropharyngeal cancer patients.

	Univariate			Multivariate		
Characteristics	HR	95% CI	P value	HR	95% CI	P value
OS						
Gender (female vs. male)	0.047	0.000–1069.263	0.550	0.000	0.000-	0.980
T classification (T3/T4 vs. T1/T2)	2.391	1.043–5.482	0.039	3.337	1.312–8.488	0.011
Primary tumor treatment (Surgery vs. CCRT)	1.689	0.504–5.664	0.391	0.607	0.123–3.000	0.540
Neck treatment (Neck dissection vs. CCRT)	2.085	0.869–5.000	0.100	2.199	0.522–9.256	0.283
Induction chemotherapy (Yes vs. No)	0.514	0.192–1.373	0.184	0.557	0.128–2.242	0.410
P16 (Positive vs. Negative)	0.165	0.035–0.772	0.009	0.177	0.031–0.917	0.041
LRFS						
Gender (female vs. male)	0.047	0.000–824.709	0.540	0.000	0.000-	0.980
T classification (T3/T4 vs. T1/T2)	2.131	0.969–4.689	0.060	3.054	1.242–7.509	0.015
Primary tumor treatment (Surgery vs. CCRT)	1.486	0.446–4.947	0.519	0.446	0.079–2.536	0.363
Neck treatment (Neck dissection vs. CCRT)	1.971	0.832–5.671	0.123	2.689	0.448–16.145	0.280
Induction chemotherapy (Yes vs. No)	0.448	0.169–1.186	0.106	0.629	0.124–3.185	0.575
P16 (Positive vs. Negative)	0.165	0.035–0.772	0.009	0.197	0.036–0.985	0.048
RRFS						
Gender (female vs. male)	0.047	0.000–563.595	0.524	0.000	0.000-	0.978
T classification (T3/T4 vs. T1/T2)	1.873	0.878–3.993	0.104	2.354	1.037–5.342	0.041
Primary tumor treatment (Surgery vs. CCRT)	1.196	0.361–3.963	0.770	0.588	0.120–2.884	0.513
Neck treatment (Neck dissection vs. CCRT)	1.522	0.648–3.573	0.335	1.284	0.270–6.115	0.754
Induction chemotherapy (Yes vs. No)	0.508	0.193–1.335	0.169	0.457	0.098–2.132	0.319
P16 (Positive vs. Negative)	0.130	0.028–0.606	0.002	0.082	0.012–0.566	0.011
DMFS						
Gender (female vs. male)	0.047	0.000–785.047	0.538	0.000	0.000-	0.979
T classification (T3/T4 vs. T1/T2)	2.389	1.080–5.287	0.032	3.307	1.289–7.157	0.011
Primary tumor treatment (Surgery vs. CCRT)	1.710	0.513–5.697	0.382	0.706	0.150–3.322	0.660
Neck treatment (Neck dissection vs. CCRT)	1.940	0.819–4.597	0.132	1.962	0.503–7.660	0.322
Induction chemotherapy (Yes vs. No)	0.572	0.216–1.515	0.261	0.643	0.167–2.485	0.522
P16 (Positive vs. Negative)	0.157	0.031–0.737	0.007	0.131	0.020–0.844	0.032

For nonoropharyngeal cancer patients, univariate and multivariate analyses for survival are summarized in Table 3. With regard to primary tumor treatment, (surgery vs. CCRT) no difference was noted in terms of OS (HR: 0.940; 95% CI: 0.247–3.571; p = 0.927), LRFS (HR: 0.780; 95% CI: 0.227–2.675; p = 0.693), RRFS (HR: 1.033; 95% CI: 0.281–3.802; p = 0.961) or DMFS (HR: 0.665; 95% CI: 0.207–2.135; p = 0.493). Neck treatment (neck dissection vs. CCRT) did not affect OS (HR: 0.444; 95% CI: 0.127–1.549; p = 0.203), LRFS (HR: 0.473; 95% CI: 0.149–1.503; p = 0.204), RRFS (HR: 0.364; 95% CI: 0.101–1.274; p = 0.114) or DMFS (HR: 0.717; 95% CI: 0.248–2.077; p = 0.540). The 2-year survival outcome in terms of OS, LRFS, RRFS, and DMFS for definitive CCRT to the neck (CCRT group) or curative neck dissection followed by adjuvant CCRT (neck dissection group) were 37.0% and 45.6% (Fig 1E), 27.8% and 45.2% (Fig 1F), 33.3% and 45.6% (Fig 1G), and 33.3% and 32.8% (Fig 1H), respectively.

**Table 3:** Univariate and multivariate analysis for survival in non-oropharyngeal cancer patients.

	Univariate			Multivariate		
Characteristics	HR	95% CI	P value	HR	95% CI	P value
OS						
Gender (female vs. male)	0.989	0.234–4.177	0.988	0.621	9,142–2.725	0.528
T classification (T3/T4 vs. T1/T2)	2.466	0.853–7.132	0.096	2.899	0.862–9.746	0.085
Primary tumor treatment (Surgery vs. CCRT)	1.139	0.548–2.368	0.727	0.940	0.247–3.571	0.927
Neck treatment (Neck dissection vs. CCRT)	0.714	0.346–1.475	0.363	0.444	0.127–1.549	0.203
Induction chemotherapy (Yes vs. No)	0.602	0.286–1.269	0.182	0.306	0.100–0.932	0.037
LRFS						
Gender (female vs. male)	0.816	0.194–3.429	0.781	0.491	0.112–2.140	0.343
T classification (T3/T4 vs. T1/T2)	2.218	0.844–5.828	0.106	2.675	0.869–8.227	0.086
Primary tumor treatment (Surgery vs. CCRT)	0.950	0.466–1.933	0.087	0.780	0.227–2.675	0.693
Neck treatment (Neck dissection vs. CCRT)	0.638	0.316–1.289	0.210	0.473	0.149–1.503	0.204
Induction chemotherapy (Yes vs. No)	0.710	0.341–1.475	0.358	0.351	0.122–1.004	0.051
RRFS						
Gender (female vs. male)	0.958	0.227–4.037	0.954	0.626	0.143–2.751	0.535
T classification (T3/T4 vs. T1/T2)	1.745	0.662–4.603	0.261	1.927	0.640–5.085	0.244
Primary tumor treatment (Surgery vs. CCRT)	1.022	0.497–2.101	0.954	1.033	0.281–3.802	0.961
Neck treatment (Neck dissection vs. CCRT)	0.660	0.324–1.342	0.251	0.364	0.101–1.274	0.114
Induction chemotherapy (Yes vs. No)	0.602	0.288–1.259	0.178	0.307	0.103–0.915	0.034
DMFS						
Gender (female vs. male)	0.814	0.194–3.415	0.778	0.582	0.134–2.525	0.470
T classification (T3/T4 vs. T1/T2)	1.700	0.699–4.136	0.242	2.044	0.735–5.687	0.171
Primary tumor treatment (Surgery vs. CCRT)	0.896	0.447–1.797	0.758	0.665	0.207–2.135	0.493
Neck treatment (Neck dissection vs. CCRT)	0.758	0.383–1.500	0.426	0.717	0.248–2.077	0.540
Induction chemotherapy (Yes vs. No)	0.769	0.372–1.591	0.479	0.440	0.155–1.245	0.122

**Fig 2:**
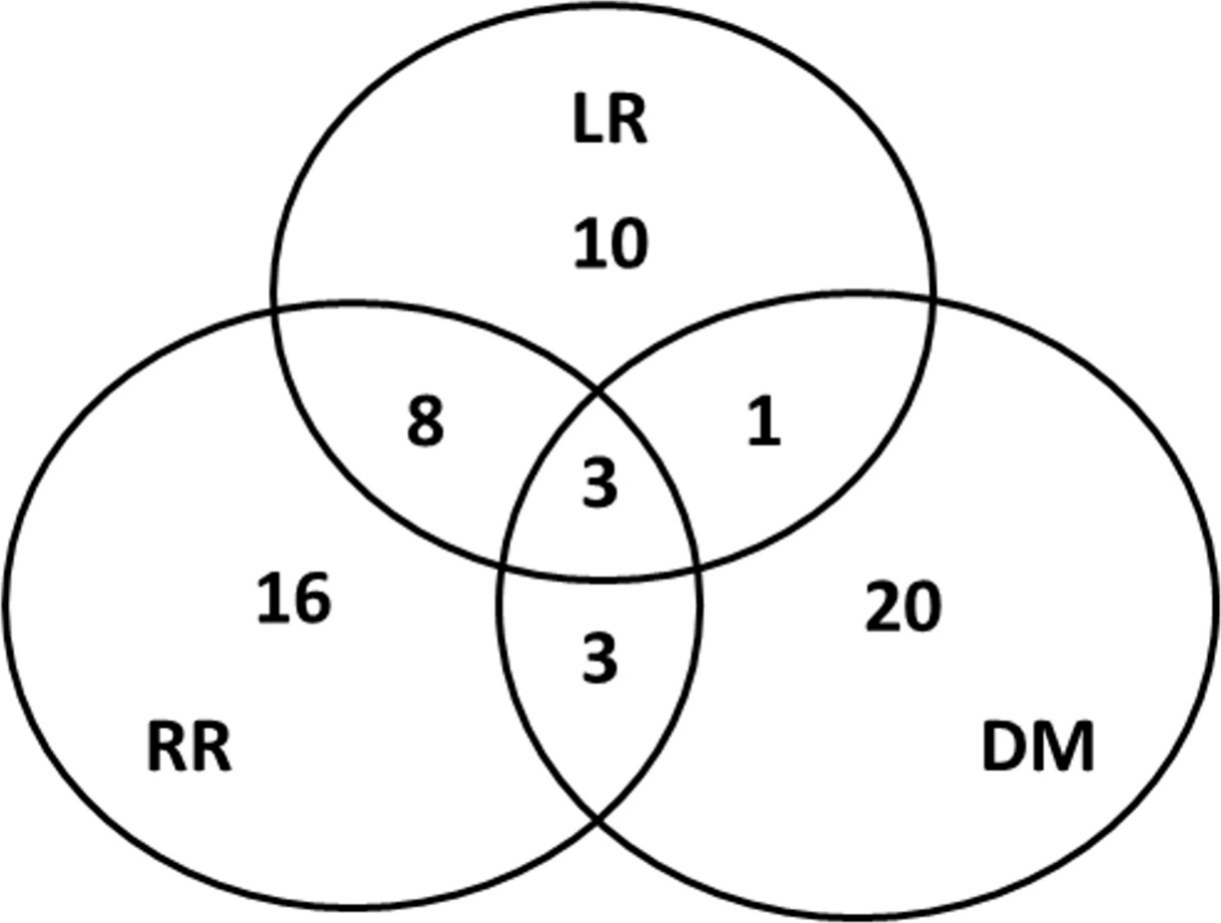
Pattern of first failure sites with numbers of patients. LR, local recurrence; RR, regional recurrence; DM, distant metastasis.

Among the 93 patients, 32 (34.4%) had disease-free recurrence at last follow-up. The first failure sites are summarized in Fig 2. In total, 30 out of the 61 patients experiencing recurrence had regional recurrence, whereas 27 had distant metastasis. Local recurrence occurred in 22 of the 61 patients.

Among our patients, acute grade ≥ 3 toxicities were observed in 82% of CCRT group and in 85% of neck dissection group. Late grade ≥ 3 toxicities were 9% and 10% for CCRT and neck dissection, respectively.

## Discussion

Studies focusing on the management of N3 head and neck patients are limited. The results of previous and current studies are summarized in Table 4. Adams et al.[8] reported outcomes for 33 N3 head and neck cancer patients treated with definitive CCRT and PET-guided neck management. Their patient cohort consisted of 25 (76%) cases of oropharyngeal; 4 (12%), nasopharyngeal; 1 (3%), laryngeal; and 1 (3%) hypopharyngeal malignancy. Overall PET CR rate was 64.5%, and subsequent nodal failure rate after PET CR was 10% (2 patients). The 3-year nodal control rate and metastasis-free survival rate for all patients were 68.6% and 59.5%, respectively. For the patients with oropharyngeal cancer, the 3-year nodal control rate and metastasis-free survival were 64.8% and 59.1%, respectively.

**Table 4:** Summary of outcomes for N3 neck cancer patients in the literature.

Study	Igidbashian et al. [9]	Karakaya et al. [10]	Adams et al. [8]	Zenga et al. [11]	Argiris et al. [12]	Corry et al. [13]	Jung et al. [14]	Ko et al. [15]	Smyth et al. [16]	Jones et al. [17]	Witek et al. [18]	Chen et al. (current study)
Study period	1998–2006	2004–2010	2005–2012	1998–2013	1991–2000	1998–2002	2000–2010	2004–2012	1989–2009	1975–2005	1991–2015	2002–2015
No. of patients	70	40	33	39	25 total N2/N3 (N3: 6 patients)	102 total N2/N3 (N3: 20 patients)	121 N2; 70 N3	4867	100	275 (119 patients with radical treatment)	36	93
Primary tumor site	56 (80%) oropharynx, 8 (11.4%) unknown, 2 (2.9%) larynx, 2 (2.9%) oral cavity, and 2 (2.9%) hypopharynx	24 (60%) oropharynx, 4 (10%) larynx, 2 (2.9%), 6 (15%) hypopharynx, 2 (5%) oral cavity, and 4 (10%) unknown	25 (76%) oropharynx, 4 (12%) nasopharynx, 1 (3%) larynx, and 1 (3%) hypopharynx	16 (41%) base of tongue, 22 (56%) tonsil, and 1 (3%) unknown	Unknown primary	3 (3%) oral cavity, 78 (76%) oropharynx, 15 (15%) hypopharynx, 6 (6%) larynx	30 (42.9%) oropharynx, 18 (25.7%) hypopharynx, 11 (15.7%) larynx, 8 (11.4%) nasopharynx, 3 (4.3%) oral cavity	425 (8.7%) oral cavity, 3275 (67.3%) oropharynx, 538 (11.1%) hypopharynx, 629 (12.9%) larynx	53% oropharynx, 7% larynx, 15% hypopharynx, 3% nasopharynx, 8% oral cavity, 4% multiple sites, 11% unknown	16 (13%) larynx, 27 (23%) hypopharynx, 30 (25%) oropharynx, 42 35%) oral cavity	67% oropharynx, 11% Unknown, 11% hypopharynx, 8% larynx, and 3% oral cavity	49 (52.7%) oropharynx, 26 (27.9%) hypopharynx, 14 (15%) oral cavity, and 4 larynx (4.3%)
Neck management	Definitive CCRT with neck dissection only for those with incomplete response	Definitive CCRT without planned neck dissection	Definitive CCRT with PET-guided management at 12 weeks	Neck dissection with or without adjuvant therapy	Definitive CCRT in 8 (32%) patients, neck dissection in 17 (64%) patients	Definitive CCRT without planned neck dissection	Definitive CCRT 32 (45.7%), neck dissection in 38 (54.3%)	Definitive CCRT 3403 (70%), neck dissection in 1464 (30%)	Definitive CCRT 76%, neck dissection in 24%	119 patients: neck dissection + adjuvant therapy	20 (56%) definitive CCRT, 8 (22%) RT alone, 8 (22%) surgery	Definitive CCRT without planned neck dissection or upfront neck dissection followed by adjuvant therapy
Overall survival	2-year at 63.0% for cCR and 79.4% and cPR-ND	3-year at 51.4%	3-year at 48.4%	5-year at 87%	N3 patients: 3-year 33%	NA for N3	N3: 5-year disease-free survival 36.3%	Propensity-adjusted median survival: 54.2 and 44.8 months for surgery and CCRT, respectively (P = 0.06).	Oropharynx: 5-year 80% for surgery, 46% for CCRT (p = 0.3); Non-oropharynx: 58% for surgery, 15% for CCRT (p = 0.02)	5yr 26.6%	5yr 30%, no difference in definitive CCRT or surgery	Oropharynx: 2-year at 57.4% for definitive CCRT and 37.5% for neck dissection. Non-oropharynx: 2-year at 37.0% for definitive CCRT and 45.6% for neck dissection
Neck control	2-year regional relapse-free survival at 87.8% for cCR patients	3-year at 69.3%	3-year nodal control rate at 68.6%	Isolated regional disease recurrence or persistence in two (5%) patients	NA	N3: 40% nodal CR rate at 12 weeks post treatment	N3: 5-year local-regional control rate 75.5%	NA	NA	NA	no difference in definitive CCRT or surgery	Oropharynx: 2-year at 50.6% for definitive CCRT and 37.5% for neck dissection. Nonoropharynx: 2-year at 33.3% for definitive CCRT and 45.6% for neck dissection
Distant failure	2-year distant disease-free survival at 67.1 for cCR and 92.6% for cPR-ND	NA	3-year metastasis-free survival at 59.5%	NA	NA	NA	N3: 5-year DM rate 60%	NA	NA	NA	no difference in definitive CCRT or surgery	Oropharynx: 2-year at 56.3% for definitive CCRT and 37.5% for neck dissection. Nonoropharynx: 2-year at 33.3% for definitive CCRT and 32.8% for neck dissection

Abbreviations: cCR, clinically complete response; cPR-ND, neck dissection after achieving cCR at the primary site and clinically partial response in the neck; NA, not available; CCRT, concurrent chemoradiotherapy

Karakaya et al. [10] reported on 40 N3 head and neck cancer patients treated with definitive CCRT. Of them, 24 (60%), 4 (10%), 6 (15%), 2 (5%), and 4 (10%) had oropharyngeal, laryngeal, hypopharyngeal, oral cavity, and unknown primary cancer, respectively. Twenty-seven (67.5%) patients achieved CR with subsequent nodal failure rate of 3/27 (11%). The 3-year overall survival and regional control in the whole cohort were 51.4% and 69.3%, respectively. Igidbashian et al.[9] reported on 70 N3 patients treated with definitive CCRT with neck dissection only for those with incomplete response. Oropharyngeal patients comprised 56 (80.0%) of the cohort. The CR rate was 26/70 (37.1%), and the 2-year regional relapse-free survival was 87.8% for patients who achieved clinical CR. Our data showed that CR rate in the neck in patients with oropharyngeal and nonoropharyngeal cancer were 31/41 (75.6%) and 12/18 (66.7%), respectively. A total of 7/31 (22.6%) patients with oropharyngeal cancer and 3/12 (25%) patients with nonoropharyngeal cancer who achieved CR in the neck after definitive CCRT had subsequent regional recurrence. In our definitive CCRT to the neck cohort, the overall 2-year RRFS rate was 45.2%, while it was 50.6% and 27.8% in patients with oropharyngeal and nonoropharyngeal cancer, respectively.

Meanwhile, Zenga et al.[11] reported the outcomes of upfront neck dissection for 39 patients with N3 human papillomavirus (HPV)-related oropharyngeal cancers. Thirty-six (90%) underwent adjuvant therapy, with 69% of them receiving adjuvant CCRT. Isolated regional disease recurrence or persistence was found in two (5%) patients. Five-year OS, disease-specific survival, and disease-free survival were 87%, 89%, and 84%, respectively. In our study, oropharyngeal cancer patients who received upfront neck dissection followed by adjuvant CCRT had 2-year OS and RRFS of 37.5% and 37.5%, respectively. The result probably reflects the effects of the combination of HPV-positive and HPV-negative oropharyngeal cancer in our cohort. In our study, specifically for HPV (+) patients, 5yr OS for CCRT and neck dissection group were 80% and 68%, respectively. In the current study, the 2-year survival outcome in terms of OS and RRFS for definitive CCRT to neck (CCRT group) or curative neck dissection followed by adjuvant CCRT (neck dissection group) was 45.6% and 45.6%, respectively.

Smyth et al.[16] analyzed 100 head and neck N3 patients. They found that for non-oropharyngeal cancer, those who underwent primary surgery (n = 14) had significantly better OS than those who had primary CCRT (n = 32, P = 0.02). Our data showed no difference between neck dissection or definitive CCRT. However, Smyth et al.[16] included 4% nasopharyngeal cancer, 8.5% multi-site primary cancer and 23% unknown primary carcinoma in non-oropharyngeal cancer. The outcomes for nasopharyngeal cancer, multi-site primary cancer and unknown primary carcinoma differ significantly from that of pure head and neck cancer squamous cell carcinoma, which might explain the difference between the 2 studies. Similar to our findings, Witek et al.[18] also showed that OS was similar between patients receiving primary surgery, radiotherapy, or chemoradiotherapy (p = 0.10). Patients with p16-positive tumors exhibited improved overall (p = 0.05).

The largest N3 study so far was conducted by Ko et al[15]. They performed retrospective analysis of 4867 patients in National Cancer Database (NCDB). After adjusting for age, sex, and Charlson/Deyo comorbidity score, race, insurance status, income, location, patient volume of treatment facilities, tumor subsite, tumor size, T classification, HPV status and radiation dose/technique by propensity score, median survival was 54.2 and 44.8 months for surgery and CCRT, respectively (P = 0.06).

Distant failure is a major failure pattern for N3 head and neck patients. Our data showed a 2-year distant metastasis-free survival of around 35–40%. Jung et al. also showed a high 5-year DM rate of 60%[14]. In this extreme high risk patients, the potential role of induction chemotherapy or chemotherapy regimen intensification should be further investigated.

As for treatment toxicities, review article and meta-analysis comparing neck dissection followed by adjuvant therapy and definitive CCRT to neck showed that no difference in grade ≥ 3 toxicities for acute (80% vs. 86%) and late toxicities (8% vs. 6%). Neck fibrosis rates of around 20% were reported for both groups[19]. For tri-modality therapy, Zenga et al.[11] showed a 5% pneumonia rate, 5% admission rate during adjuvant for acute kidney injury, and 8% other side effects (surgical site infection, pharyngocutaneous fistula, sepsis related to a gastrostomy tube complication). Witek et al.[18] showed that acute toxicities were similar between surgery and definitive CCRT. Sixty-eight percent of patients in the neck dissection (68.4% v 68.0%; p = 0.98) groups required a feeding tube for a median of 6 months (range 2–42 months versus 3–33 months; p = 0.59). Unplanned hospitalization within 6 months from diagnosis was similar between surgery and CCRT groups (27.8% versus 36.0%; p = 0.57).

Our study showed that the survival outcomes in terms of OS, LRFS, RRFS, or DMFS for N3 oropharyngeal and nonoropharyngeal cancer patients treated with bimodality definitive CCRT to the neck did not differ from those treated with trimodality curative neck dissection followed by adjuvant CCRT. The present study showed that even for bulky N3 neck, bimodality definitive CCRT to the neck without planned neck dissection can be the treatment of choice. However, this study has some limitations. During the study period, PET-CT was not routinely performed in our institution. Response evaluation in this study was done by both clinically local examination and MRI. Complete response was defined by undetectable primary tumor or shrinkage of neck lymph nodes to less than 1cm in short axis on T2 weighted and T1 weighted with contrast medium MRI. However, it is not unusual to detect post-treatment mass, either as fibrosis or true residual tumors. This study had a 25% ultimate regional failure rate among CR patients. Adams et al.[8] and Karakaya et al.[10] reported a 10–11% subsequent nodal failure rate after CR. No routine use of PET in our study may be one of the reasons for higher nodal failure rate for differently defined CR patients. With more widespread PET-CT implementation in head and neck cancer, a more accurate staging, target definition, and treatment response evaluation can be achieved[20]. This study may also have treatment modality selection bias due to its retrospective nature. Adjusted Kaplan Meier analysis was used to account for unequal balance in factors. For oropharyngeal cancer, after adjusting for gender, T classification, primary tumor treatment (Surgery vs. CCRT), induction chemotherapy (Yes vs. No) and P16 status, there were no significant differences in terms of neck treatment (neck dissection vs. CCRT) for OS (p = 0.379), LRFS (p = 0.775), RRFS (p = 0.510) and DMFS (p = 0.989). Although adjusted Kaplan Meier analysis might handle unequal balance in factors to some extent, limited numbers in subgroups was one of the weakness.

## Conclusion

In summary, N3 neck patients treated with definitive CCRT can achieve similar outcomes to those treated with upfront neck dissection followed by adjuvant CCRT. Bimodality definitive CCRT can be the primary treatment of choice for this group of patients with poor prognosis. Cautions should be made to avoid overtreatment for this group of patients.

## Supporting information

S1 Fig Treatment summary. (TIF)

S2 Fig Survival curve. (a) OS, (b) LRFS, (c) RRFS, and (d) DMFS for all patients.
(TIFF)
