# Supplementary figures and images for: Outcome of bimodality definitive chemoradiation does not differ from that of trimodality upfront neck dissection followed by adjuvant treatment for >6 cm lymph node (N3) head and neck cancer
Source: PLoS One. 2019 Dec 3;14(12):e0225962. doi: 10.1371/journal.pone.0225962 (PMC6890260; doi:10.1371/journal.pone.0225962)

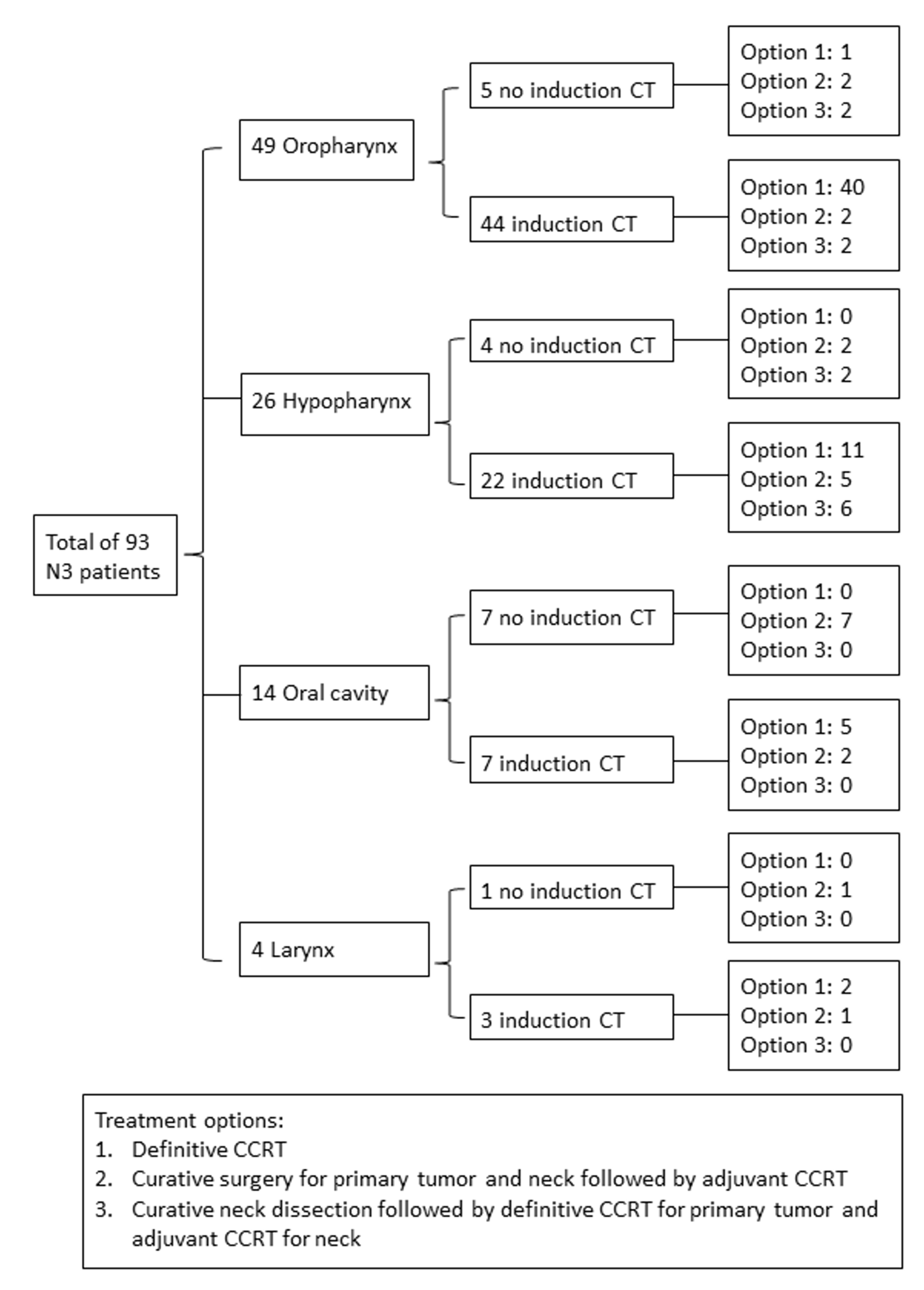

Supplement: S1 Fig — (TIF) [file pone.0225962.s001.tif]

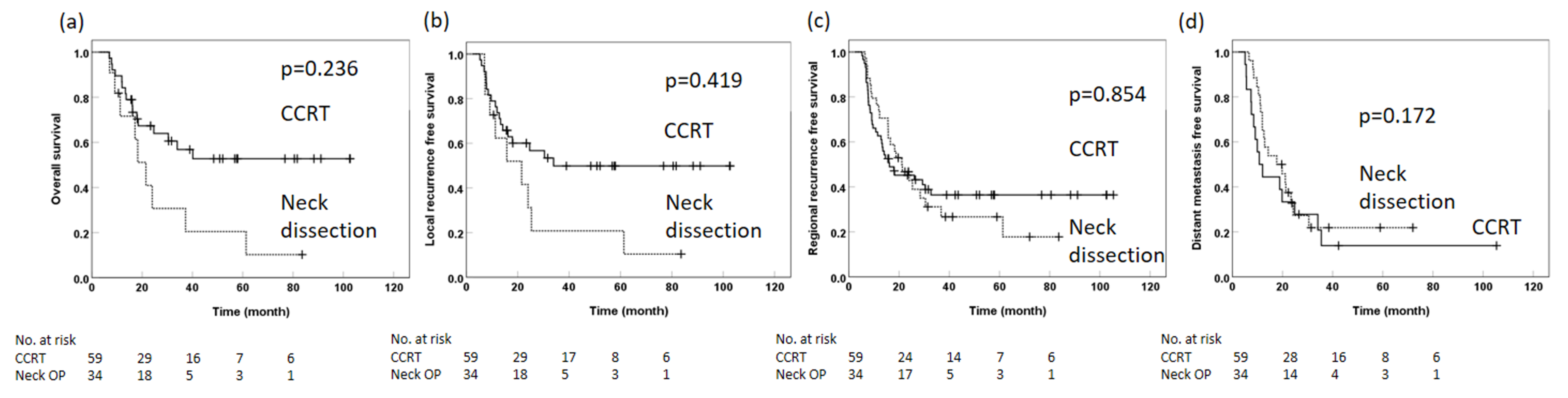

Supplement: S2 Fig — (a) OS, (b) LRFS, (c) RRFS, and (d) DMFS for all patients. (TIFF) [file pone.0225962.s002.tiff]
